# Relevant genetic differentiation among Brazilian populations of *Anastrepha fraterculus* (Diptera, Tephritidae)

**DOI:** 10.3897/zookeys.540.6713

**Published:** 2015-11-26

**Authors:** Mosè Manni, Kátia Manuela Lima, Carmela Rosalba Guglielmino, Silvia Beatriz Lanzavecchia, Marianela Juri, Teresa Vera, Jorge Cladera, Francesca Scolari, Ludvik Gomulski, Mariangela Bonizzoni, Giuliano Gasperi, Janisete Gomes Silva, Anna Rodolfa Malacrida

**Affiliations:** 1 Department of Biology & Biotechnology, University of Pavia, Via A. Ferrata 9, 27100 Pavia, Italy; 2 Departamento de Ciências Biológicas, Universidade Estadual de Santa Cruz, Rodovia Jorge Amado km 16, 45650-000 - Ilhéus, Bahia, Brazil; 3 Instituto Genética EA Favret, Instituto Nacional de Tecnología Agropecuaria, 1686 Hurlingham, Provincia Buenos Aires, Argentina; 4 Consejo Nacional de Investigaciones Científicas y Técnicas, Av. Rivadavia 1917, Buenos Aires, Argentina; 5 Facultad de Agronomía y Zootecnia, Universidad Nacional de Tucumán, Florentino Ameghino s/n (4000), Tucumán, Argentina

**Keywords:** *Anastrepha fraterculus*, microsatellites, population genetic differentiation, morphotypes

## Abstract

We used a population genetic approach to detect the presence of genetic diversity among six populations of *Anastrepha fraterculus* across Brazil. To this aim, we used Simple Sequence Repeat (SSR) markers, which may capture the presence of differentiative processes across the genome in distinct populations. Spatial analyses of molecular variance were used to identify groups of populations that are both genetically and geographically homogeneous while also being maximally differentiated from each other. The spatial analysis of genetic diversity indicates that the levels of diversity among the six populations vary significantly on an eco-geographical basis. Particularly, altitude seems to represent a differentiating adaptation, as the main genetic differentiation is detected between the two populations present at higher altitudes and the other four populations at sea level. The data, together with the outcomes from different cluster analyses, identify a genetic diversity pattern that overlaps with the distribution of the known morphotypes in the Brazilian area.

## Introduction

The South American fruit fly *Anastrepha fraterculus* Wiedemann (Diptera: Tephritidae) belongs to the *fraterculus* group, which comprises a total of 34 formally described species (Norrbom et al. 2012) that can be distinguished only by minor morphological characters. The major diagnostic character for species within this group is the aculeus apex, which shows a large degree of intraspecific variation (Araujo and Zucchi 2006) as a result of genetic and environmental factors (Aluja 1994, Smith-Caldas et al. 2001).

The nominal species *Anastrepha fraterculus* is widely distributed from the Rio Grande Valley in northern Mexico to central Argentina, infesting over 100 hosts (Norrbom 2004), thus being a species of major economic importance in Brazil and other countries in South America (Steck 1991, Steck 1999, Zucchi 2008). Its distribution in South America was thought to be in two broad and unconnected bands along the east coast and the western and northern edges of the continent with a hiatus comprising the Amazon basin (Steck 1999, Malavasi et al. 2000). Yet, recent collections have recorded this species in the Brazilian Amazon in the states of Amapá, Pará, Tocantins, and Maranhão infesting 10 different hosts (Zucchi et al. 2011).

*Anastrepha fraterculus* has long been reported to show extensive morphological variation along its geographic distribution (Lima 1934, Stone 1942, Steck 1991, 1999). In his taxonomic review of the genus *Anastrepha*, Stone (1942) stated “it is possible that it will eventually be found to represent a complex of species rather than a single one”. Since then, a good deal of research has documented and concluded that the nominal species *Anastrepha fraterculus* actually comprises an unresolved complex of cryptic species. Evidence comes from studies on morphological variation (Steck 1999, Hernández-Ortíz et al. 2012, for review), multivariate morphometric analyses (Hernández-Ortíz et al. 2004, 2012), differences in host use (Steck 1999, for review), behaviour (Yamada and Selivon 2001, Selivon et al. 2005, Vera et al. 2006, Vaníčková et al. 2015), the presence and degree of reproductive isolation (Devescovi et al. 2014 and references therein), and genetic analyses (Silva and Barr 2008, for review). However, the actual number of species within the *Anastrepha fraterculus* complex and their distribution is yet to be elucidated.

Genetic studies performed on *Anastrepha fraterculus* populations so far have revealed the following putative biological entities based on geography: an Andean lineage (Steck 1991), a Mexican species (Steck 1991, Smith-Caldas et al. 2001, Barr et al. 2005 and the morphometric studies of Hernández-Ortíz et al. 2004, 2012), a Guatemalan lineage (Smith-Caldas et al. 2001), a second Venezuelan species (Smith-Caldas et al. 2001), a Peruvian lineage (Steck 1991), and three Brazilian species (Morgante et al. 1980, Smith-Caldas et al. 2001, Selivon et al. 2005, and Ruiz et al. 2007a, 2007b) (Silva and Barr 2008, for review). In addition, morphometric studies revealed that Mexican populations formed a single morphotype, which was distinct from South American populations (Hernández-Ortíz et al. 2004). Hernández-Ortíz et al. (2004) also identified an Andean morphotype (one population from Colombia) and a Brazilian morphotype comprising two populations from Brazil (states of São Paulo and Santa Catarina) and a population from Argentina (Tucumán). A study using isozymes, karyotypes, morphometry, and crossings on populations of *Anastrepha fraterculus* from Brazil recognized two species, *Anastrepha* sp.1 aff. *fraterculus* and *Anastrepha* sp.2 aff. *fraterculus* (Selivon et al. 2005). More recently, a multivariate morphometric analysis comprising 32 *Anastrepha fraterculus* populations identified seven distinct morphotypes: a Mexican morphotype, a Venezuelan morphotype, an Andean morphotype, a Peruvian morphotype, and three Brazilian morphotypes (Brazilian 1, Brazilian 2, and Brazilian 3) (Hernández-Ortíz et al. 2012). Although Brazilian populations of the nominal species *Anastrepha fraterculus* most likely comprise at least three morphotypes, published studies are based mostly on samples from the south-eastern region, while very few populations from other regions of the country have been examined.

Previous genetic studies using DNA sequencing of mitochondrial genes from different populations suggested that both the *fraterculus* group and the *Anastrepha fraterculus* complex have a recent evolutionary history, and thus molecular markers with a higher power of resolution were required to help understand the specific/subspecific differentiation within and between populations of this group (McPheron et al. 1999, Smith-Caldas et al. 2001). Microsatellites are a class of highly polymorphic molecular markers widely distributed in the genome of eukaryotes that can be useful to clarify such patterns of gene flow and to identify the spatial locations of genetic discontinuities (population boundaries) in studies of species complexes (Chambers and MacAvoy 2000, Barbará et al. 2007, Aketarawong et al. 2014). Within the Tephritidae, microsatellites have been successfully developed and applied for several *Bactrocera* species (Shearman et al. 2006; Aketarawong et al. 2006, 2007, Augustinos et al., 2008, Virgilio et al. 2010; Drew et al. 2011), for *Rhagoletis cerasi* L. (Augustinos et al. 2011), for a few *Ceratitis* species (Bonizzoni et al. 2001, 2004, Meixner et al. 2002, Baliraine et al. 2003, 2004, Silva et al. 2003, Delatte et al. 2013, Virgilio et al. 2013), for *Anastrepha suspensa* (Loew) (Fritz and Schable 2004, Boykin et al. 2010), and for *Anastrepha obliqua* (Macquart) (Islam et al. 2011). Microsatellites have only recently been isolated in *Anastrepha fraterculus* (Lanzavecchia et al. 2014) and have proven useful for the analysis of population dynamics and differentiation across the distribution range of this polymorphic species.

From an applied perspective, the correct identification of populations and species is an important step in the implementation of biologically-based control methods such as the Sterile Insect Technique (SIT) against this fruit fly complex (Silva and Barr 2008), which represents a serious constraint for fruit production in South America and a hindrance to the export of fresh fruit from regions where it occurs. Currently, control measures for this pest species rely solely on the use of insecticide cover or bait sprays. Therefore, there is demand for the development of the SIT as it would benefit South American countries such as Argentina, Brazil, and Peru (Cladera et al. 2014). However, the application and efficiency of species-specific control methods such as the SIT are critically dependent on the correct identification of the target pest populations and the understanding of the spatial distribution of the pest species, thus the correct delimitation of species within the *Anastrepha fraterculus* complex is paramount.

This paper is centred on the assessment of genetic diversity among *Anastrepha fraterculus* populations from distinct geographic regions across Brazil, most likely belonging to at least three distinct morphotypes (Silva et al. unpubl. data). For this purpose we used highly informative SSR markers, which may capture the presence of eventual differentiative processes across the genome in different populations.

## Methods

**Figure 1.**
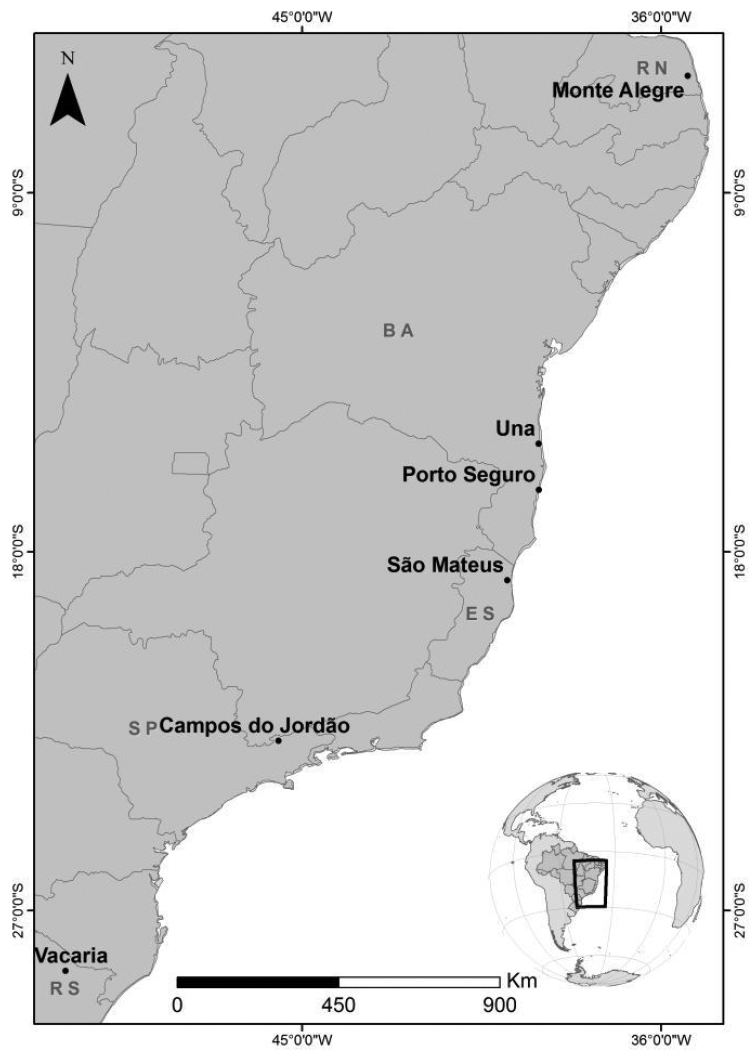
Map of the collected samples.

Six populations from three regions across Brazil were sampled from 2007 to 2013 (Table 1, Figure 1). In the Northeastern region, the populations from Monte Alegre (State of Rio Grande do Norte) and from Una and Porto Seguro (State of Bahia) were sampled. In the Southeastern region, samples from São Mateus (State of Espírito Santo) and from Campos do Jordão (State of São Paulo) were examined. In the Southern region, the population from Vacaria (State of Rio Grande do Sul) was sampled. For each locality, flies emerging from fruits collected from different trees were considered. Adult females were identified as *Anastrepha fraterculus* by Dr. Elton L. Araujo (UFERSA), Dr. Keiko Uramoto (USP), Dr. Miguel Francisco Souza Filho (Instituto Biológico) and Dr. Roberto A. Zucchi (USP) using the aculeus shape following Zucchi (2000) (Table 1). Voucher specimens were deposited at the insect collection of the Escola Superior de Agricultura “Luiz de Queiroz”, USP, Piracicaba, SP, and at the Universidade Estadual de Santa Cruz, Ilhéus, BA, Brazil. According to the classification of Hernández-Ortíz et al. (2012), the flies collected in Una (BA) can be classified as Brazilian morphotype 3, while those from Vacaria (RS) and Campos do Jordão (SP) as Brazilian 1 (Silva et al. unpublished data). For the samples from Monte Alegre, Porto Seguro and São Mateus, no clear-cut information is available to assign them to a specific morphotype.

**Figure 2.**
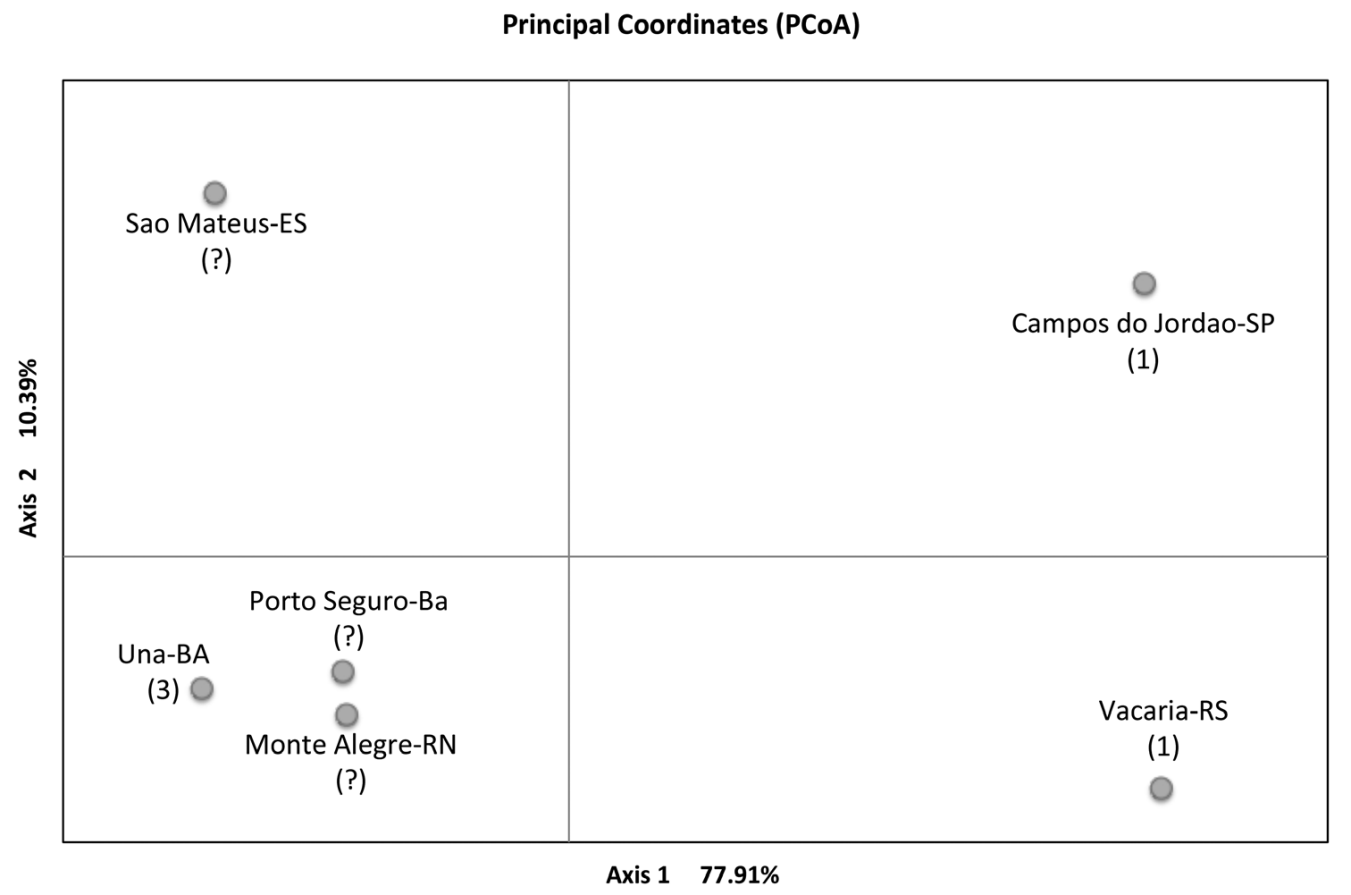
Two-dimensional plot of Principal Coordinate Analysis (PCoA) based on similarity matrix derived from *Anastrepha fraterculus* microsatellites data. The Morphotype classification (Hernández-Ortiz et al. 2012), relative to each sample is also reported.

**Table 1.** Field collected samples of *Anastrepha fraterculus* Brazilian populations.

States	Sample site	Morphotype*	Host	Coordinate	Elevation
**Rio Grande do Norte (RN)**	Monte Alegre	?	Guava	6.0678W; 35.3322S	51.816m
**Bahia (BA)**	Una	3	Guava	15.2933W; 39.0753S	27.737m
**Bahia (BA)**	Porto Seguro	?	Guava	16.4497W; 39.0647S	48.768m
**Espírito Santo (ES)**	São Mateus	?	Araçá	18.7161W; 39.8589S	35.966m
**São Paulo (SP)**	Campos do Jordão	1	Raspberry	22.7394W; 45.5914S	1627.9m
**Rio Grande do Sul (RS)**	Vacaria	1	Guava	28.5122W; 50.9339S	970.79m

* The Morphotype classification is based on Hernández-Ortiz et al. (2012)

### Microsatellite analysis

A total of 171 *Anastrepha fraterculus* individuals collected from the above mentioned populations were assessed for their SSR variability. DNA was extracted from three legs of each single fly using the “DNeasy Blood & Tissue” kit (Qiagen, Valencia, CA) following the standard DNeasy protocol. DNA samples were screened using the following ten microsatellite loci: AfD4, AfD105, AfA7, AfA112, AfA115, AfA120, AfA122, AfA117, AfA10, and AfC103 (Lanzavecchia et al. 2014). Allele scoring was performed using an automated ABI PRISM 310 Genetic Analyser (Applied Biosystem) following Aketarawong et al. (2006).

### Data analysis

The mean number of alleles (na) and mean null allele frequency (*A*n) (non-amplifying alleles due to changes in the primer binding regions), expected and observed heterozygosity were estimated using GENEPOP version 4.0.7 (Raymond and Rousset 1995) for each population. Deviation from the Hardy-Weinberg equilibrium and linkage disequilibrium, together with their critical levels after the sequential Bonferroni test (Rice 1989), were tested using GENEPOP version 4.0.7 (Raymond and Rousset 1995). The allelic Polymorphic Information Content (PIC) was derived using CERVUS (Kalinowski et al. 2007).

Microsatellite Analyzer (MSA) (Dieringer and Schlötterer 2003) was applied to estimate the pairwise *F*st values among populations (Weir and Cockerham 1984). The statistical significance of each *F*st value was assessed by comparing the observed values with the values obtained in 10,000 matrix permutations. Spatial analyses of molecular variance were investigated using SAMOVA 2.0 (Dupanloup et al. 2002). This approach identifies groups of populations that are genetically homogeneous and maximally differentiated from each other without the constraint of being geographically close. The method requires the *a priori* definition of the number of groups (*K*) of populations that exist and generates *F*-statistics (*F*_SC_, *F*_ST_ and *F*_CT_) using an AMOVA approach. Different numbers of groups (*K*) were tested, and a simulated annealing procedure permitted the identification of the composition of each of the *K* groups that maximizes the *F*_CT_ index (proportion of total genetic variance due to differences between groups). The program was run for two to five groups (*K* = 2 to *K* = 5) each time with the simulated annealing process repeated 100 times, starting each time with a different partition of the population samples into the *K* groups. The analysis of molecular variance (AMOVA) was carried out using ARLEQUIN software version 3.11 (Excoffier et al. 2006). Principal Coordinate Analysis (PCoA) in the program GenAIEx 6.5 (Peakall and Smouse 2012) was applied to identify the relationships among populations on the basis of their allele frequencies.

## Results

### SSR variability

The variability estimates describing the suitability of the ten SSR loci (AfD4, AfD105, AfA7, AfA112, AfA115, AfA120, AfA122, AfA117, AfA10, and AfC103) for detecting the presence of differentiation among the *Anastrepha fraterculus* populations are shown in Table 2. The number of alleles per locus across populations ranges from 7 to 18 with a mean of 12.9, and the mean frequency of null alleles across the loci is generally low (0.04–0.08). Moreover, the Polymorphic Information Content (PIC) estimate for each locus ranges from 0.44 (A10a) to 0.88 (A120a), and the across loci average is 0.74, suggesting that this set of loci is informative for population analyses.

**Table 2.** Microsatellite variability detected across the six Brazilian *Anastrepha fraterculus* populations.

Locus	na	Min–Max	PIC
D4a	7	2–5	0.56
D105a	15	5–11	0.72
A7a	13	7–11	0.83
A112a	18	8–12	0.82
A115a	14	7–12	0.80
A120a	15	7–13	0.88
A122a	12	5–9	0.74
A117a	11	6–9	0.77
A10a	13	2–11	0.44
C103a	11	7–9	0.80
Mean	12.9	5.6–10.2	0.74

na, mean number of alleles; Min–Max, minimum and maximum number of alleles; PIC, polymorphic information content.

Tests for Hardy-Weinberg equilibrium (HWE) using Fisher’s exact test with the sequential Bonferroni correction (Rice 1989) revealed that the populations conformed to Hardy–Weinberg equilibrium (HWE) at most loci. The very few observed locus/populations combinations that were not in HWE were not concentrated at any locus or in any population. Significant linkage disequilibrium was not detected between genotypes at the ten loci. As no evidence of linkage disequilibrium between loci was assessed, these 10 loci can be considered to be independent.

### Population variability and differentiation

**Figure 3.**
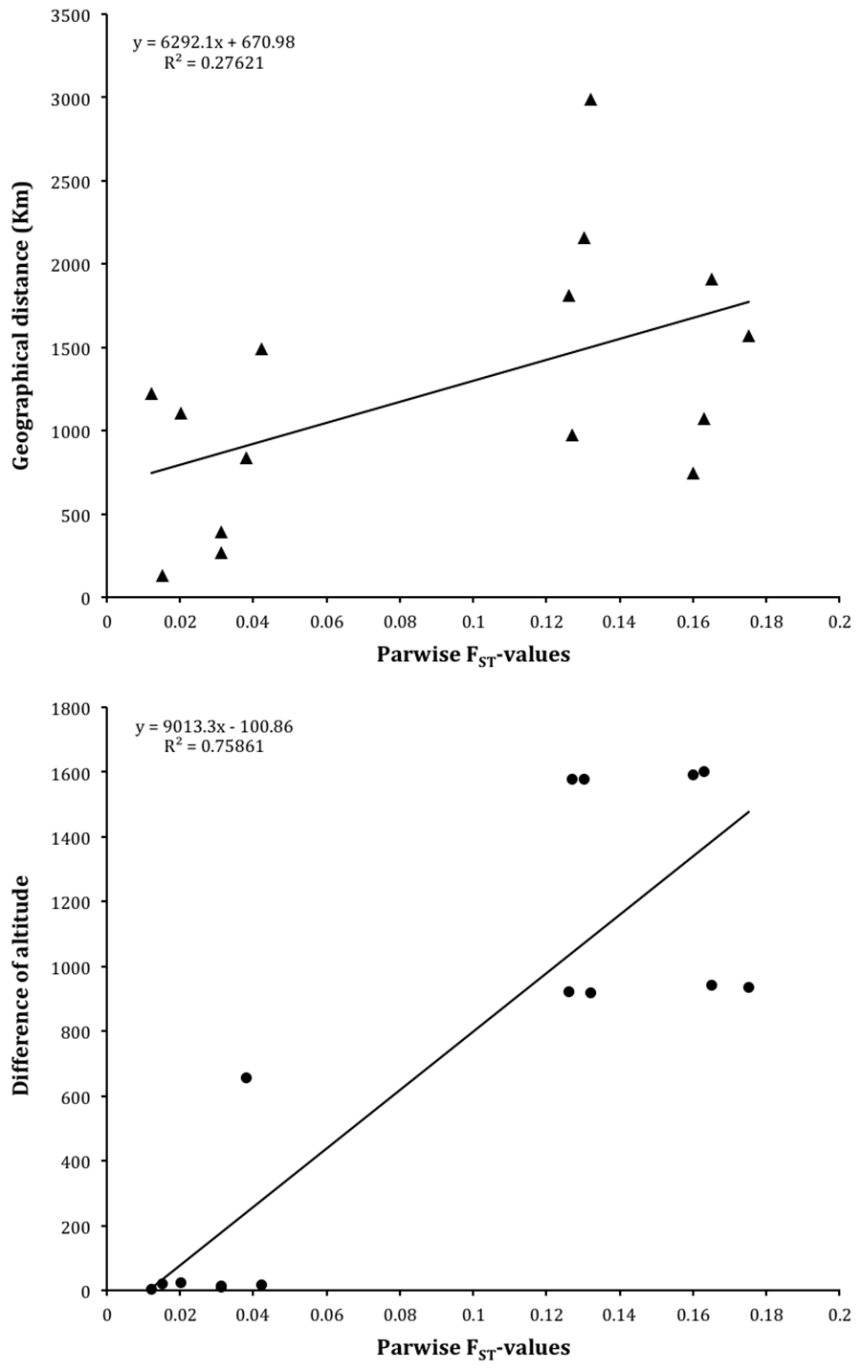
Correlation of *FST* values with the geographic distances (upper plot) and altitude differences (bottom plot) among the 6 Brazilian samples of *Anastrepha fraterculus*.

An estimate of variability distribution in and among the six tested populations (AMOVA) indicates that 90% of the variation occurs within populations while only about 10% of total variation is detected among populations. Indeed as shown in Table 3, the intrapopulation genetic variability is similar across the six samples. As a second step, the simulated annealing approach based on the SAMOVA algorithm was applied to identify the presence of genetically homogeneous groups across the considered Brazilian populations. For this, the spatial analyses of molecular variance was performed without constraints for geographic composition of the groups. As observed in Table 4 no great differences in the *F*_CT_ values were observed when we increased the group number (K): four of the five simulated groupings (2, 4, and 5) produced non-significant *F*_CT_ values. This implies that the molecular variance due to differences between populations within each group is weak, confirming the AMOVA data. Only in one case did we observe an *F*_CT_ estimate which statistically maximized the differences between groups. This is the grouping configuration which splits the six populations into three groups: i) Una, Porto Seguro and São Mateus, ii) Monte Alegre, iii) Campos do Jordão and Vacaria, (*F*_CT_ = 0.182, P = 0.015). These data indicate that the mountain populations of Campos do Jordão (state of São Paulo) and Vacaria (state of Rio Grande do Sul) are genetically homogeneous and differentiated from the group of the three coastal populations (Una, Porto Seguro and São Mateus), and from the other more distant coastal population of Monte Alegre. The pairwise *F*_ST _estimates (Table 5) confirm the presence of differentiation between the group of the two mountain populations and the coastal populations which in turn share a certain degree of genetic relatedness. Principal Coordinate Analysis (PCoA) was performed to better clarify the genetic relations among the six populations. The first two axes explain a relatively high amount of the genetic variation (88%). The first axis (77.91%) separates Campos do Jordão and Vacaria from the other populations. The second axis (10.39%) mainly differentiates São Mateus from the group of Una, Porto Seguro, and Monte Alegre, but also Campos do Jordão from Vacaria. It is interesting that there is a certain correspondence of the genetic grouping with the known morphotype classification (Hernández-Ortiz et al. 2012). The molecular variance represented by the first axis separates the populations on the basis of both geographical distance and altitude. On this basis, we attempted to disentangle the effect of geographical distance and altitude on the genetic differentiation of Campos de Jordão and Vacaria. The plots in Figure 3 show the correlations between pairwise *F*_ST_ values with the differences in altitude (Figure 3A) and the geographic distance (Figure 3B), respectively. The correlation results clearly indicate that the difference in altitude has a greater impact (r^2^ = 0.76 vs r^2^ = 0.28) on genetic differentiation of mountain Vacaria and Campos do Jordão, than on the remaining samples belonging to the coastal plain populations.

**Table 3.** Genetic variability of wild populations of *Anastrepha fraterculus* from different geographical regions in Brazil estimated using 10 SSRs.

	na	He	Ho	*F_IS_*
**Una-BA**	7,7	0,63	0,54	0,14
**Porto Seguro-BA**	8,2	0,70	0,67	0,02
**Monte Alegre-RN**	7,5	0,68	0,57	0,20
**São Mateus-ES**	6,3	0,66	0,66	0,08
**Campos do Jordão-SP**	8,3	0,71	0,61	0,13
**Vacaria-RS**	8,5	0,72	0,62	0,12

na, mean number of alleles; He, expected heterozygosity; Ho, observed heterozygosity; *F_IS_*, fixation index.

**Table 4.** Spatial Analysis of Molecular Variance (SAMOVA) for different population partitions.

**Number of groups (*K*)**	***F*_CT_**	***P***	**Population partition**
2	0.195	0.062	(Una, Porto Seguro, Monte Alegre, São Mateus), (Campos do Jordão, Vacaria)
3	0.182	0.015	(Una, Porto Seguro, São Mateus), (Monte Alegre), (Campos do Jordão, Vacaria)
4	0.190	0.050	(Una, Porto Seguro, São Mateus), (Monte Alegre), (Campos do Jordão), (Vacaria)
5	0.196	0.068	(Una, Porto Seguro), (São Mateus), (Monte Alegre), (Campos do Jordão), (Vacaria)

**Table 5.** Pairwise-F_ST_ values among 6 population samples of *Anastrepha fraterculus* as derived from Microsatellite Analyser (Dieringer and Schlotterer 2003).

	Una-BA	Porto Seguro-BA	Monte Alegre-RN	São Mateus-ES	Campos do Jordão-SP	Vacaria-RS
**Una-BA**	-					
**Porto Seguro-BA**	0.015 ^ns^	-				
**Monte Alegre-RN**	0.020	0.012 ^ns^	-			
**São Mateus-ES**	0.031	0.031	0.042	-		
**Campos do Jordão-SP**	0.163	0.127	0.130	0.160	-	
**Vacaria-RS**	0.165	0.126	0.132	0.175	0.038	-

Some of the values are not significantly different from zero at P > 0.05 (ns)

## Discussion

With this paper we initiated, in the polymorphic *Anastrepha fraterculus* complex, an analysis of the underpinning genetic architecture and its interaction with correlated ecological, biological and morphological traits. In this context, we have used microsatellite markers to perform a genetic analysis of populations from a complex ecological area such as Brazil.

Although the chromosomal location of the considered SSR loci remains unknown, the assessed linkage equilibrium between them suggests they are statistically independent and that their variability patterns might reflect genome-wide patterns across populations. The six considered ecogeographic populations are here represented by highly polymorphic samples, which reflect a high degree of intrapopulation genetic diversity. Indeed only 10% of the total variability (AMOVA) is represented by the differences between the six geographic populations, while greater variability was found within populations.

The spatial analysis of genetic diversity indicates that the levels of diversity among the six populations vary significantly on an eco-geographical basis, as indicated by SAMOVA and PCoA data. More than by geographical distance, the genetic differentiation is influenced by altitude. The multivariate analysis of ten microsatellites depicts a structural pattern, which clearly separates populations on climatic distribution both on latitudinal and altitudinal basis. Particularly, altitude seems to represent a differentiating adaptation, as the main genetic differentiation is that detected between the populations present at higher altitudes (Campos de Jordão and Vacaria) and those populations from sea level. Genetic divergence between populations from low and high altitude areas has already been observed for populations within the *Anastrepha fraterculus* complex using isozymes (Morgante et al. 1980, Steck 1991, Selivon et al. 2005) and mtDNA (Steck and Sheppard 1993, Santos 1999, McPheron et al. 1999, Smith-Caldas et al. 2001, Barr et al. 2005). Steck (1991) concluded that the strong differences in allelic frequency between lowland and Andean populations of *Anastrepha fraterculus* in Venezuela was due to the fact that they actually represent two genetically distinct species albeit morphologically indistinguishable. These allopatric populations can be subject to divergent selection in response to ecological factors that change over large geographical scales such as altitude. Altitude may act as a barrier to gene flow as levels of life history divergence between high- and low-altitude populations can be correlated with levels of post-zygotic reproductive isolation (Orr and Smith 1998).

One interesting observation, which arises from our data, is that the observed structure of Brazilian populations is entangled with the presence of morphotypes. The actual number of these entities and their respective geographic range are questions that remain to be further elucidated. At the moment three different morphotypes are identified in Brazil (Hernández-Ortíz et al. 2012). As it appears clearly, the PCoA analysis depicts a genetic differentiative pattern that overlaps with the distribution of the known morphotypes. Now the open questions are: 1) is the observed population differentiation contributing to the underpinning genetic architecture of the morphotypes associated to these populations? and 2) do morphotypes track environmental variability? In retrospect, a further aim is to clarify the evolutionary relationships between populations, ecotypes, and morphotypes.

## Conclusion

The population genetic approach, in addition to improving our knowledge of the underpinning genetic architecture of the *Anastrepha fraterculus* complex, is also important from an applied perspective. The overall level of genetic variability and the presence of differentiation that we detected among the Brazilian populations of *Anastrepha fraterculus* constitute an important contribution for any potential future application of SIT for the control of populations of this fruit fly pest in Brazil.
